# What is the real impact of acute kidney injury?

**DOI:** 10.1186/1471-2369-15-95

**Published:** 2014-06-21

**Authors:** Michael Bedford, Paul E Stevens, Toby WK Wheeler, Christopher KT Farmer

**Affiliations:** 1 Kent Kidney Research Group, East Kent Hospitals University NHS Foundation Trust, Kent and Canterbury Hospital, Ethelbert Road, Canterbury, Kent CT1 3NG, UK

**Keywords:** AKI, Incidence, Impact, Outcomes, General hospital

## Abstract

**Background:**

Acute kidney injury (AKI) is a common clinical problem. Studies have documented the incidence of AKI in a variety of populations but to date we do not believe the real incidence of AKI has been accurately documented in a district general hospital setting.

The aim here was to describe the detected incidence of AKI in a typical general hospital setting in an unselected population, and describe associated short and long-term outcomes.

**Methods:**

A retrospective observational database study from secondary care in East Kent (adult catchment population of 582,300). All adult patients (18 years or over) admitted between 1^st^ February 2009 and 31^st^ July 2009, were included. Patients receiving chronic renal replacement therapy (RRT), maternity and day case admissions were excluded. AKI was defined by the acute kidney injury network (AKIN) criteria. A time dependent risk analysis with logistic regression and Cox regression was used for the analysis of in-hospital mortality and survival.

**Results:**

The incidence of AKI in the 6 month period was 15,325 pmp/yr (adults) (69% AKIN1, 18% AKIN2 and 13% AKIN3). In-hospital mortality, length of stay and ITU utilisation all increased with severity of AKI. Patients with AKI had an increase in care on discharge and an increase in hospital readmission within 30 days.

**Conclusions:**

This data comes closer to the real incidence and outcomes of AKI managed in-hospital than any study published in the literature to date. Fifteen percent of all admissions sustained an episode of AKI with increased subsequent short and long term morbidity and mortality, even in those with AKIN1. This confers an increased burden and cost to the healthcare economy, which can now be quantified. These results will furnish a baseline for quality improvement projects aimed at early identification, improved management, and where possible prevention, of AKI.

## Background

Acute kidney injury (AKI) is a common clinical problem characterised by an abrupt decline in kidney function, ranging from a small rise in serum creatinine (SCr) to anuric kidney failure requiring renal replacement therapy (RRT). AKI may either be present on admission to hospital, or develop during the course of admission. The many aetiologies and risk factors for AKI are well described [1-4], as are the short and long term consequences [1,2,4-7].

In the last decade the definition of AKI has been standardised, refined and adopted in clinical research [6,8] leading to improved understanding of the epidemiology of AKI and a realisation of its potential health economics impact [9].

A number of studies have documented the incidence of AKI in a variety of populations [9-20] but to date we do not believe that the real incidence of AKI has been accurately documented in a district general hospital setting. The aims of this study were therefore to (i) use the acute kidney injury network (AKIN) definition to describe the real incidence of AKI in a typical general hospital setting in an unselected patient population, (ii) describe the associated short and long-term outcomes, (iii) describe the health and social care consequences of AKI.

## Methods

### Patient population

Ethical approval for this study was obtained from Kent Research Ethics Committee (ref 10/H1101/89). All adult patients (18 years or over) admitted to East Kent Hospitals University NHS Foundation Trust (EKHUFT) between 1^st^ February and 31^st^ July 2009 were included. Time of entry to the cohort was the date of admission for each patient. EKHUFT comprises 3 general hospitals with a total of 1250 inpatients beds serving a defined population of approximately 744,400 people (582,300 adults) in the geographical area of East Kent in the southeast peninsula of England [21]. Patients were followed up until the 31^st^ March 2011. Patients receiving chronic renal replacement therapy (RRT) (including dialysis and renal transplantation), maternity admissions and day case admissions were excluded from the analyses.

### Data extraction

Data were extracted from the EKHUFT data warehouse. This data warehouse stores patient demographics and all patient episodes, including primary diagnosis and co-morbidity for each episode. Unique patient identifiers were used to link the data warehouse with the pathology database.

AKI was defined by the AKIN criteria using the lowest SCr in the 12 months prior to the date of hospital admission as the reference after the method of Lafrance et al. [22]. In cases where there were no pre-hospitalisation values and the follow up SCr (lowest in the 12 months following discharge) was lower than the peak in the study admission, the follow up creatinine was used as the reference SCr. The assumption was made that if SCr had improved following admission by greater than 26.4 μmol/L, then the admission must have involved an AKI (UK Renal Association, Acute Kidney Injury Clinical Practice Guideline) [23].

The peak creatinine during the inpatient stay was used to define the stage of AKI.

### Independent variables

Patient demographics (to determine age and eGFR calculations), postcode (to determine deprivation score), co-morbidity, and primary diagnosis were extracted. Both co-morbidity and primary diagnosis were coded for each hospital episode on the data warehouse using ICD-10 codes. For primary diagnoses the ICD-10 group was extracted for each admission (Additional file 1: Table S1). For co-morbidity (secondary diagnoses), validated coding algorithms from Quan et al., [24] with further validated algorithms for diabetes [25] and hypertension, were used to determine a modified Charlson co-morbidity score for each patient. The number of admissions and outpatient appointments in the 12 months prior to a patient admission were also recorded. From the baseline pathology data, the baseline chronic kidney disease (CKD) stage was defined for each patient, using the baseline creatinine (lowest creatinine in the 12 months prior to admission), or the post-discharge nadir creatinine was used for the subset of patients without a pre-hospitalisation creatinine.

### Outcomes

Mortality, hospital length of stay (LOS), intensive care LOS, and change in residence resulting from admission were recorded. Date of death and 30 day re-admission rates were also recorded wherever relevant. The date of death was obtained from the Patient Master Index (PMI) on the hospital patient administration system (PAS). Where a patient died in hospital this field was populated using the discharge details of the patient’s episode and was therefore validated at the point the patient was discharged as ‘died in hospital’. Where a patient died following discharge the PAS PMI record was updated via a weekly report from the Open Exeter national system which provides the date of death for any patient recently deceased [26]. Data on LOS, intensive care LOS, re-admission, and place of discharge were complete, as recorded on the hospital PAS.

All admissions during the recruitment and follow up periods were extracted. AKI stage was calculated for all admissions until the end of the follow up in order to inform the survival analysis.

Data were also extracted from the renal data system (Renal Plus, CHI) and from the intensive care database to determine whether patients in this cohort received RRT during admission, and whether they were still dependent on RRT 90 days post discharge. Patients who received RRT (often in ITU) but did not meet the creatinine criteria for AKIN 3, were upgraded to AKIN 3 in line with the specifications of the AKIN criteria.

### Statistical methods

Patient level demographic summaries were performed, considering a single observation per patient. For patients with more than one admission with AKI data were summarised at the time of the admission with their highest AKI stage where there was a valid reference SCr. For patients who had no valid AKI recordings over the course of the study, data from the first admission was used in the analysis.

Normally distributed data were summarised as the mean and standard deviation. Continuous data not normally distributed were summarised by median and inter-quartile range, or the percentage of values in each category for categorical variables.

Three of the continuous variables, Charlson co-morbidity score, number of previous admissions in the previous 12 months, and number of outpatient appointments in the previous 12 months all had a very highly skewed distribution. So that outlying values were not overly influential, these three variables were categorised for analysis.

Chosen outcomes of interest were mortality, LOS, intensive therapy unit (ITU) utilisation, and increase in care following discharge. Regression analyses were performed to determine the impact of AKI on each outcome. Variables used in the regression model, and thought to be confounders were age, gender, primary diagnosis, modified Charlson co-morbidity score, stage of chronic kidney disease (CKD). We also added admission from residential or nursing care, deprivation index, hospital admissions and outpatient appointments in the last 12 months. The analyses were performed in three stages. In the first analysis, the effect of AKI upon each outcome was examined (an unadjusted analysis). The second analysis was age and gender adjusted and the third analysis was multiply adjusted, including the above variables.

For primary diagnosis in the regression model, specifically for elective admissions there were diagnosis groups with too few events. Therefore elective admissions were set as the reference and emergency admissions split by ICD-10 group for primary diagnosis.

Logistic regression was used for the analysis of in-hospital mortality and Cox regression for survival analysis. A time dependent risk analysis for survival was employed to allow adjustment for multiple admissions during the study and follow up period.

Analysis of LOS, which was highly skewed, was performed using negative binomial regression. The analysis of LOS was performed at the admission level in the recruitment period, and hence patients may have contributed to the analysis several times during the recruitment period. To allow for the correlation between repeat LOS values from the same patients a multilevel approach was employed, equivalent to fitting a random-effects model for subjects in addition to the fixed effects model.

In order to assess the social impact of AKI the change in residence related to the admission was assessed. An increase in care from home prior to admission to hospital, to residential or nursing care on discharge, was classified as an increase in care on discharge. This assessment was performed by stage of AKI.

## Results

### Population characteristics and AKI

**Figure 1:**
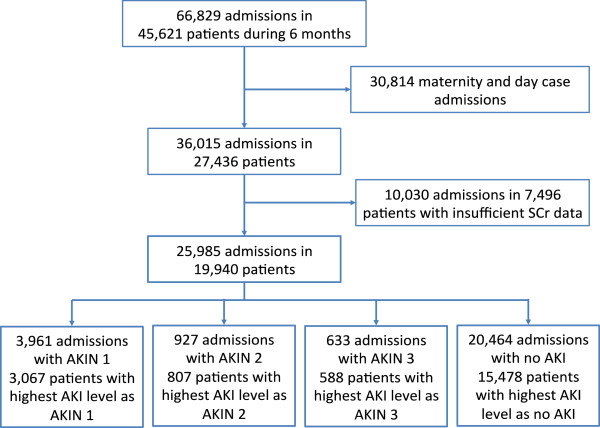
Derivation of the study population.

During the 6 month recruitment period there were 66,829 admissions in 45,621 adult patients (Figure 1). After exclusion of maternity and day case admissions there were 36,015 admissions in 27,436 patients (79.1% of patients had 1 admission during the 6 month recruitment, 14.6% had 2 admissions, 4.1% had 3 and 2.2% had 4 or more). Overall, there were 10,030 admissions in 7,496 patients with insufficient SCr data to define AKI. Of these 42.9% were elective admissions and 57.1% were non-elective, the majority had a LOS of 0–2 days (see below). There were 20,464 admissions with no AKI and 5,521 admissions with AKI (8.8% of all admissions and 15.3% of non-maternity and non-day case admissions). Of these, 3,961 admissions had AKIN 1, 927 admissions AKIN 2, and 633 admissions AKIN 3. Of the 5,521 admissions with AKI, 4064 had AKI on admission (73.6%) and 531 of 633 admissions with AKIN 3 (83.9%) had AKI on admission.

Of the 36,015 admissions, baseline creatinine data in the 12 months prior to admission was available in 31,435 (87%). In the remaining 4,580 admissions the lowest creatinine in the 12 months following discharge (in survivors) was used as the baseline serum creatinine. In these 4,580 admissions, 7.2% had AKIN 1, 1.4% AKIN 2 and 1.3% AKIN 3. This is in comparison to admissions in which a baseline from the 12 months following discharge was not used, in which 11.5% had AKIN 1, 2.7% AKIN 2 and 1.8% AKIN 3. In admissions culminating in mortality baseline creatinine data was obtainable in 1209/1379 (88%).

Overall, only 455/5521 admissions with AKI (8.2%) involved the calculation of a baseline using the lowest creatinine in the 12 months following discharge.

For descriptive statistics patients without sufficient SCr data (“no AKI info”) are reported in the results but only those patients with valid SCr data sufficient to define AKI were included in the regression analyses. Patients with insufficient data to define AKI were younger, had less co-morbidity and shorter LOS than other patients (Tables 1 and 2).

**Table 1:** Summaries of mean age, gender, deprivation and co-morbidity at a patient level, only considering admissions during the recruitment period, and for multiple admissions per patient during the recruitment period selecting the patient’s admission with the highest AKI stage

**Variable**	**No AKI**	**AKIN 1**	**AKIN 2**	**AKIN 3**	**No AKI info**
	**(n = 15,478)**	**(n = 3,067)**	**(n = 807)**	**(n = 588)**	**(n = 7,496)**
Age - Mean (SD)	62.0 (20.3)	74.2 (16.3)	76.1 (14.7)	72.5 (15.7)	54.2 (21.0)
Age: 18-39	17.1%	5.1%	3.6%	4.4%	29.0%
40-59	23.7%	11.3%	8.9%	16.0%	28.3%
60-79	36.9%	38.2%	37.3%	40.7%	29.5%
80+	22.3%	45.5%	50.2%	39.0%	13.2%
Male Sex - %	45.1%	52.2%	45.0%	49.8%	45.8%
Deprivation - Median (IQR)	17.4 (11.8 27.0)	17.2 (11.8, 25.8)	17.3 (11.8, 25.8)	17.2 (11.9, 26.9)	17.2 (11.7, 26.7)
AIDS - %	0.1%	0.1%	0.1%	0.0%	0.0%
Malignancy - %	6.2%	11.5%	14.0%	16.7%	4.8%
CHF - %	2.6%	10.4%	13.9%	11.6%	1.0%
CPD - %	12.8%	17.0%	16.1%	17.4%	8.5%
Cerebrovascular disease - %	7.3%	13.5%	12.3%	11.2%	3.4%
Dementia - %	3.2%	6.7%	8.2%	7.0%	1.9%
Diabetes - %	10.3%	20.2%	18.7%	23.8%	6.0%
Hemiplegia. - %	1.3%	1.8%	1.4%	1.5%	0.5%
Hypertension - %	27.2%	39.%	39.3%	39.0%	15.5%
MI - %	3.0%	5.0%	6.0%	3.9%	0.7%
Solid tumour - %	2.0%	3.2%	4.8%	4.4%	0.9%
Liver disease - %	0.9%	1.8%	3.0%	6.1%	0.5%
PVD - %	2.1%	5.4%	6.2%	4.6%	1.0%
Peptic ulcer - %	0.6%	1.2%	1.7%	1.9%	0.4%
Renal disease - %	1.7%	11.2%	16.4%	22.3%	1.1%
Rheumatic disease - %	2.3%	3.9%	3.1%	4.1%	1.1%
CKD - no data	0%	0%	0%	0.7%	34.4%
no CKD	84.8%	61.9%	62.1%	68.2%	58.0%
CKD stage 3a	10.0%	19.1%	20.1%	15.0%	5.0%
CKD stage 3b	4.0%	13.1%	12.1%	10.2%	2.0%
CKD stage 4	1.0%	5.3%	5.5%	2.6%	0.5%
CKD stage 5	0.2%	0.5%	0.2%	3.4%	0.1%
Charlson ≤ 0 - %	58.0%	31.9%	25.8%	23.3%	74.5%
1-10 - %	25.9%	29.4%	30.5%	30.1%	17.4%
11 + =%	16.2%	38.8%	43.7%	46.6%	8.2%

Chronic pulmonary disease (CPD), chronic heart failure (CHF), myocardial infarction (MI), peripheral vascular disease (PVD), acquired immunodeficiency syndrome (AIDS).

**Table 2:** A summary of the length of stay for: all patients, those who died in hospital, and those who survived to hospital discharge, split by AKI stage

**Statistic**	**No AKI**	**AKIN 1**	**AKIN 2**	**AKIN 3**	**No AKI info**
	**(n = 20,464)**	**(n = 3,961)**	**(n = 927)**	**(n = 633)**	**(n = 10,030)**
All patients					
Mean (SD)	4.5 (10.5)	9.7 (14.6)	12.3 (16.0)	14.9 (18.5)	2.3 (9.8)
Median (IQR)	2 (0, 5)	5 (1, 12)	7 (3, 15)	9 (4, 20)	1 (0, 2)
Died in hospital					
Mean (SD)	11.1 (14.4)	11.8 (16.3)	10.0 (11.9)	10.3 (12.2)	13.5 (29.1)
Median (IQR)	6 (2, 14)	6 (2, 15)	6 (2, 14)	6 (2, 14)	5 (1, 15)
Survived to hospital discharge					
Mean (SD)	4.4 (10.4)	9.5 (14.5)	13.0 (17.1)	17.2 (120.5)	2.1 (8.8)
Median (IQR)	1 (0, 5)	5 (1, 11)	8 (3, 15)	11 (5, 22)	1 (0, 2)

Length of stay is summarised as a continuous variable, and then additionally split into categories.

The crude incidence of AKI in the 6 month period was 3,067 patients with AKIN 1, 807 AKIN 2, and 588 AKIN 3. In total, 4,462 patients from a catchment population of approximately 582,300 adults experienced AKI during the 6 month recruitment period, assuming the same incidence for the remaining 6 months of the year from a population of 582,300 this represents an incidence of 15,325 per million (adult) population per year (pmp/yr).

Co-morbidity as evidenced by the Charlson co-morbidity score was over represented in patients with AKI, and increased with AKI stage (Table 1). Deprivation was not related to AKI stage.

### Renal Replacement Therapy (RRT)

Only 77 patients of the 588 patients with AKIN 3 (13.1%) received RRT. Of these, 16 remained on RRT 90 days following discharge (2.7% of AKIN 3). A further 4 patients who experienced AKIN 3 in their index admission (admission with highest AKI stage during the recruitment period) who did not require RRT during that admission, subsequently required chronic RRT within 90 days of discharge. There were also 2 patients with AKIN 1, 2 patients with AKIN 2 and 1 patient with no AKI info who did not require RRT in the index admission but subsequently required chronic RRT within 90 days of discharge. In total 25 patients were on chronic RRT at 90 days.

### Survival analyses

**Figure 2:**
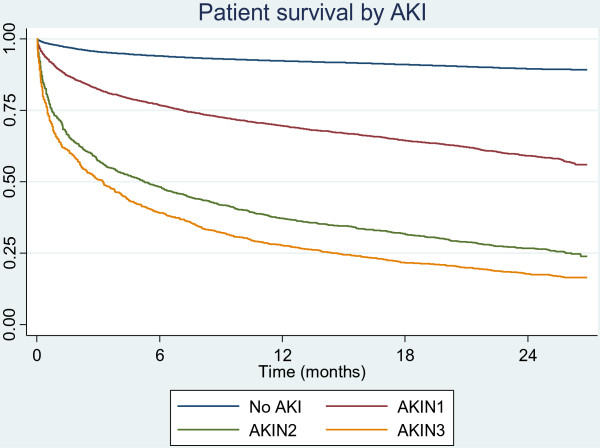
**Kaplan-Meier survival by stage of AKI.** Note that the AKI groups are based on ‘upgraded’ AKI risk.

Throughout follow up survival was related to AKI stage, (Table 3, Figure 2). In the upgraded risk analysis, after 12 months 92% of patients who had no AKI were still alive, in comparison to 28% of patients who experienced AKIN 3 (Figure 2).

**Table 3:** A summary of the survival estimates at 6-month intervals along with corresponding confidence intervals

**Variable**	**No AKI**	**AKIN 1**	**AKIN 2**	**AKIN 3**
6 m survival (95% CI)	0.94 (0.94, 0.94)	0.77 (0.75, 0.78)	0.48 (0.45, 0.52)	0.39 (0.35, 0.43)
12 m survival (95% CI)	0.92 (0.92, 0.93)	0.70 (0.68, 0.71)	0.37 (0.34, 0.40)	0.28 (0.25, 0.31)
18 m survival (95% CI)	0.91 (0.91, 0.92)	0.65 (0.63, 0.66)	0.32 (0.29, 0.35)	0.22 (0.19, 0.25)
24 m survival (95% CI)	0.90 (0.89, 0.90)	0.59 (0.58, 0.61)	0.27 (0.24, 0.29)	0.18 (0.16, 0.20)

Note that the AKI groups are based on ‘upgraded’ AKI risk. If a patient experiences a subsequent admission during follow-up with a higher stage of AKI, they will be upgraded at that point to the higher group.

Increasing severity of AKI was associated with increased risk of death and shorter survival even after multiple adjustment, AKIN 1 almost doubling the risk of death and AKIN 2 and 3 increasing the risk of death almost 3.8-fold and 5.5-fold respectively compared to those with no AKI (Table 4).

**Table 4:** Regression analyses examining the association between severity of AKI and survival, in-hospital mortality, LOS, ITU utilisation, increase in care and readmission

		**Risk of death**	**In-hospital mortality**	**ITU transfer**	**Increase in care**	**Hospital re-admission**	**Relative length of stay**	**Relative ITU length of stay**
**Model**	**Stage of AKI**	**Hazard ratio (95% CI)**	**Odds ratio (95% CI)**	**Odds ratio (95% CI)**	**Odds ratio (95% CI)**	**Odds ratio (95% CI)**	**Ratio (95% CI)**	**Ratio (95% CI)**
1	No AKI	1	1	1	1	1	1	1
	AKIN 1	4.85 (4.51, 5.21)	4.29 (3.68, 5.01)	2.36 (1.90, 2.93)	2.71 (2.17, 3.38)	1.93 (1.75, 2.13)	1.90 (1.84, 1.97)	1.38 (1.13, 1.68)
	AKIN 2	12.0 (11.0, 13.1)	16.8 (13.5, 21.1)	4.72 (3.36, 6.61)	3.71 (2.56, 5.38)	2.25 (1.83, 2.76)	2.58 (2.43, 2.75)	1.54 (1.17, 2.01)
	AKIN 3	15.6 (14.2, 17.1)	24.7 (18.8, 32.3)	23.8 (16.4, 34.6)	2.27 (1.36, 3.81)	2.09 (1.61, 2.72)	3.07 (2.85, 3.30)	2.25 (1.85, 2.73)
2	No AKI	1	1	1	1	1	1	1
	AKIN 1	3.11 (2.89, 3.35)	2.98 (2.53, 3.52)	2.63 (2.11, 3.28)	1.61 (1.29, 2.01)	1.69 (1.53, 1.87)	1.68 (1.62, 1.74)	1.43 (1.17, 1.74)
	AKIN 2	7.54 (6.89, 8.25)	13.5 (10.5, 17.5)	5.43 (3.88, 7.61)	2.07 (1.43, 2.97)	2.00 (1.63, 2.46)	2.22 (2.09, 2.36)	1.56 (1.20, 2.04)
	AKIN 3	11.6 (10.6, 12.7)	25.2 (18.6, 34.5)	23.9 (16.6, 34.4)	1.56 (0.93, 2.60)	1.94 (1.49, 2.53)	2.72 (2.53, 2.92)	2.27 (1.88, 2.76)
3	No AKI	1	1	1	1	1	1	1
	AKIN 1	1.89 (1.74, 2.04)	2.41 (1.99, 2.91)	2.76 (2.20, 3.46)	1.33 (1.06, 1.67)	1.42 (1.29, 1.57)	1.52 (1.47, 1.58)	1.39 (1.14, 1.69)
	AKIN 2	3.81 (3.46, 4.18)	12.1 (8.84, 16.5)	6.03 (4.58, 8.51)	1.49 (1.02, 2.16)	1.50 (1.23, 1.83)	1.88 (1.77, 2.00)	1.42 (1.07, 1.87)
	AKIN 3	5.49 (4.97, 6.06)	26.3 (17.8, 38.8)	22.4 (15.5, 32.2)	1.07 (0.64, 1.80)	1.54 (1.20, 1.99)	2.16 (2.01, 3.32)	2.18 (1.77, 2.68)

Model 1. Unadjusted. Model 2. Adjusted for age and gender. Model 3. Adjusted for age, gender, primary diagnosis, modified Charlson co-morbidity score, stage of chronic kidney disease (CKD), admission from residential or nursing care, deprivation index, hospital admissions and outpatient appointments in the last 12 months. All values are statistically significant, with p values < 0.001. The outcomes are defined as follows: ITU transfer - a patient being transferred to and spending any time in ITU during their hospital stay; Increase in Care - a patient being admitted from home and being discharged to residential or nursing care; Hospital Re-admission – a patient being re-admitted to hospital within 30 days following discharge; Relative Length of Stay – the ratio of length of stay in comparison to the length of stay of a patient without AKI; Relative ITU Length of Stay – the ratio of ITU length of stay (in those patients who went to ITU) in comparison to the length of stay in ITU of a patient without AKI.

### In-hospital mortality

Overall, 1,379 (3.8%) of 36,015 hospital admissions in the recruitment period resulted in an in-hospital mortality.

Only 2.0% of patients without AKI died in hospital compared with 8.1%, 25.6% and 33.3% of patients with AKIN 1, 2 and 3 respectively. AKI severity was significantly associated with in-hospital mortality even after multiple adjustment, the likelihood of mortality increasing 2.4 fold with AKIN 1 and 12 and 26 fold with AKIN 2 and 3 respectively compared to patients with no AKI (Table 4).

### Length of stay

In those patients who died in hospital LOS prior to death averaged 10.0-13.5 days irrespective of AKI (Table 2). In those surviving to leave hospital LOS was associated with severity of AKI, ranging from a mean LOS of 4.4 days in patients without AKI, to 17.2 days in patients with AKIN 3. Compared to those with no AKI after multiple adjustment LOS was 1.5, 1.9 and 2.2-fold greater in those with AKIN 1, AKIN 2 and AKIN 3 respectively (Table 4).

### Intensive Therapy Unit (ITU) utilisation

ITU utilisation increased with increasing AKI severity; 3.9%, 6.8% and 21.6% of patients with AKIN 1, 2 and 3 respectively were admitted to ITU, compared with 1.8% of patients without AKI. Intensive care LOS also increased with severity of AKI from a mean of 3.0 (SD 7.0) days in patients without AKI, to 4.4 (SD 7.8), 4.5 (5.4) and 7.3 (8.0) days in patients with AKIN stage 1, 2 and 3 respectively. After multiple adjustment, AKI severity was again associated with ITU utilisation. Patients were 2.8, 6 and 22 fold more likely to be transferred to ITU with AKIN stage 1, 2 and 3 respectively compared to patients without AKI. In patients who went to ITU their length of stay in ITU was 37%, 35%, and 111% longer in patients with AKIN stage 1,2 and 3 respectively compared to patients without AKI.

### Increase in care

A greater proportion of patients with AKI (4.5% AKIN 1, 5.7% AKIN 2 and 3.7% AKIN 3) had an increase in care on discharge in comparison to patients without AKI (1.9%). Although having an episode of AKI conferred a greater risk of increase in level of care post-discharge there was no association with severity of AKI (Table 4).

### Hospital readmission

Having an episode of AKI was also associated with an increase in hospital readmission within 30 days compared with those without AKI (Table 4), although this did not associate with severity of AKI.

## Discussion

### Summary of main findings

The incidence of AKI in an adult population reported here, 15,325 pmp/yr (10,534 pmp/yr with AKIN 1, 2,772 pmp/yr with AKIN 2 and 2,020 pmp/yr with AKIN3), is significantly higher than previous estimates reported in the literature, [20] and is likely to be closer to the real incidence in the population. The reasons for the higher incidence we report here are several. This is an unselected in-hospital population; there is increased testing of creatinine due to heightened awareness; the laboratory service in East Kent comprehensively covers the catchment population; in general because of the geography of our catchment area all patients in the area are admitted to one of our three hospital sites; our population is older in comparison to the United Kingdom average; and finally, use of the the La France methodology will also increase the reported incidence.

In this current study we have clearly demonstrated that patients with AKI, even after correcting for age, gender, co-morbidity, and CKD, have an increase in morbidity and mortality both in the short and long term in comparison to patients without AKI. These outcomes also hold true for small changes in SCr (AKIN 1). In comparison with patients with no AKI those with AKIN 1 had a 52% longer hospital stay, a 2.8-fold increased risk of admission to ITU, a 39% longer ITU stay (in those who went to ITU), and a 2.4-fold greater in-hospital mortality. Furthermore, patients with AKIN 1 had twice the long term risk of death, a 33% higher likelihood of an increase in care, and a 42% higher risk of re-admission within 30 days. In those patients with AKIN 3 (the subject of the NCEPOD report) [27] hospital LOS doubled, there was a 22 times higher risk of admission to ITU and ITU LOS was also doubled, consistent with national data from the Intensive Care National Audit and Research Centre [28]. Acute RRT support was required in 13.1% of patients with AKIN 3. Hospital mortality was 26-fold greater and in those surviving to leave hospital there was a 5.5-fold increased risk of subsequent death. Patients with AKIN 3 had a 7% higher risk of requiring an increase in care and had a 54% higher risk of readmission within 30 days than patients with no AKI. Overall, 0.45% of patients with AKI and 3.40% of patients with AKIN 3 subsequently required chronic RRT.

As the time of entry into the cohort was the date of admission for each patient there is the possibility of reverse causality, for example a patient who has a longer length of stay may have a greater risk exposure to the development of AKI. However in this cohort, of the 5521 admissions with AKI, 4064 (73.6%) already had AKI on admission.

### Strengths and weaknesses of study

The population-based analysis reported here considers all patients admitted in a general hospital setting in the United Kingdom during a 6 month period. The catchment population for this cohort is from East Kent in the South East Coast of England. Incomparison to the wider population in England East Kent has an older population (mean age 42 years compared to the national mean age of 39) but with fewer ethnic minorities (6.3% of Black and Ethnic minority compared with 14.6% nationally) [21]. Nevertheless, we believe that data linkages between the pathology, hospital data warehouse and renal systems have enabled us to come closer to the real incidence and outcomes of AKI managed in-hospital than any study published in the literature to date.

This study is a retrospective database study and clearly has limitations. Key to the definition of AKI is knowledge of pre-morbid kidney function (baseline SCr) and the threshold value of SCr from which change is measured (reference SCr). The importance of baseline SCr is in the determination of pre-existing CKD and this value should be based on SCr values available > 3 months prior to the index event. The reference SCr should be ideally be the lowest SCr recorded within 90 days of the event to distinguish this value from the baseline SCr. However, practically in many cases there may be either few or no pre-hospitalisation SCr values making distinction between baseline and reference SCr impossible. This is an area that requires further guidance and consensus from the international community and various strategies have been suggested including varying the baseline/reference creatinine from admission to 365 days prior [22], taking the average of values between 7–365 days prior to admission [29], back calculating reference SCr for missing values from an assumed MDRD glomerular filtration rate of 75 ml/min/1.73 m^2^[30], and (most recently) a method employing multiple imputation using known comorbidity strengthened by factoring in the lowest admission SCr [31]. For simplicity we chose to use the lowest SCr in the 12 months prior to the acute rise to define AKI. It may be that by doing this we have included patients with progressive CKD and defined them as AKIN 1. However, as Lafrance et al. demonstrated and our data confirms, patients with AKIN 1 using this methodology still have a significantly increased likelihood of a specific adverse outcome occurring compared to patients with no AKI [22].

The lowest serum creatinine in the 12 months following discharge was utilised to categorise AKI (for those without pre-hospitalisation creatinine) in 8.2% of admissions with AKI. We acknowledge that the assumption that AKI was present if serum creatinine improved following admission by greater than 26.4 μmol/L may not always be correct but use of this methodology was only necessary in 8% of those categorised as having AKI. The incidence of AKI in admissions utilising a post discharge baseline (9.9%) was less than in those where pre-admission creatinine data was available (16.1%).

We cannot be certain that none of the patients with insufficient SCr data experienced AKI. These patients were significantly younger and had less co-morbidity than those with sufficient SCr data and either had one or no SCr result prior to, or following hospital admission. Survivors (9,830 of 10,030) were also short stay patients (LOS 0–2 days) and were therefore unlikely to have sustained any degree of AKI. The 200 patients in this group who did not survive the hospital admission had a mean LOS of 13.5 days, lack of baseline SCr data precluded derivation of AKI status in these patients. This also raises the issue of possible ascertainment bias, that sicker patients may have more creatinine tests, increasing the probability of detecting AKI.

Co-morbidity data was extracted from the hospital data warehouse using validated algorithms, however this still relies on the accuracy of coding of clinical episodes which may not necessarily be correct. This also applies to the analysis of increase in care on discharge which replies on the accurate coding on the PAS at time of discharge.

While the statistical models used in this analysis have accounted for multiple confounders identified in the literature to date there is always the possibility that there may be other confounders hitherto unknown.

Finally, despite our estimates of the incidence of AKI in a typical general hospital setting being the highest to date, EKHUFT does not provide cardiothoracic, liver or burns services and our reported incidence of AKI may still be an under-estimation of the total population incidence.

## Conclusions

This data comes closer to the real incidence and outcomes of AKI managed in-hospital than any study published in the literature to date. Nine percent of all admissions and 15 percent of non-maternity and non-day case admissions to hospital sustained an episode of AKI with increased subsequent short and long term morbidity and mortality, even in those with AKIN1. What this study adds to existing knowledge is data enabling a much more accurate assessment of the overall impact of AKI on the healthcare economy. We provide data concerning hospital and intensive care mortality, LOS, readmission and RRT usage. We also detail the rate of RRT after longer term follow up and the social care impact in terms of increased level of care in those surviving an episode of AKI. These increased adverse outcomes from AKI confer an increased burden and cost to the healthcare economy. The data we have presented will enable this cost to be quantified and will furnish a baseline for quality improvement projects aimed at early identification, improved management, and where possible prevention, of AKI.

It has been suggested that milder forms of AKI defined by creatinine criteria may simply represent a marker of general system pathology and multi organ dysfunction, not specifically related to kidney injury per se. Whether this is true or not, AKI defines a group of patients whose outcomes are poor, both in the short and long term, who are sub-optimally managed, and who should represent a focus for patient safety improvement.

With the international agreement on the definition of AKI and its validation in clinic research, it has become clearer how important the effective management and prevention of AKI is. Agreed definitions have provided a comparable platform for the audit of AKI and its management and outcomes, both in hospital and in the community.

## Competing interests

None of the authors of this manuscript have a conflict of interest. The results presented in this paper have not been published previously in whole or part, except in abstract format.

## Authors’ contributions

MB PS CK all contributed to conception and design, interpretation of data, drafting and revising of the manuscript. TW contributed to conception and design, and extraction of data. All authors have given final approval of the manuscript version to be published.

## Pre-publication history

The pre-publication history for this paper can be accessed here:

http://www.biomedcentral.com/1471-2369/15/95/prepub

## Supplementary Material

Additional file 1: Table S1 Primary diagnoses used in the analysis, by ICD-10 group. Click here for file

## References

[B1] Mehta RL Pascual MT Soroko S Savage BR Himmelfarb J Ikizler TA Paganini EP Chertow GM Program to Improve Care in Acute Renal Disease Spectrum of acute renal failure in the intensive care unit: the PICARD experience Kidney Int 2004 66 4 1613 1621 15458458 10.1111/j.1523-1755.2004.00927.x

[B2] Metnitz PG Krenn CG Steltzer H Lang T Ploder J Lenz K Le Gall JR Druml W Effect of acute renal failure requiring renal replacement therapy on outcome in critically ill patients Crit Care Med 2002 30 9 2051 2058 12352040 10.1097/00003246-200209000-00016

[B3] Liangos O Wald R O’Bell JW Price L Pereira BJ Jaber BL Epidemiology and outcomes of acute renal failure in hospitalized patients: a national survey Clin J Am Soc Nephrol 2006 1 1 43 51 17699189 10.2215/CJN.00220605

[B4] Uchino S Kellum JA Bellomo R Doig GS Morimatsu H Morgera S Schetz M Tan I Bouman C Macedo E Gibney N Tolwani A Ronco C Beginning and Ending Supportive Therapy for the Kidney (BEST Kidney) Investigators Acute renal failure in critically ill patients: a multinational, multicenter study JAMA 2005 294 7 813 818 16106006 10.1001/jama.294.7.813

[B5] Levy MM Macias WL Vincent JL Russell JA Silva E Trzaskoma B Williams MD Early changes in organ function predict eventual survival in severe sepsis Crit Care Med 2005 33 10 2194 2201 16215369 10.1097/01.ccm.0000182798.39709.84

[B6] Bellomo R Ronco C Kellum JA Mehta RL Palevsky P Acute Dialysis Quality Initiative workgroup Acute renal failure - definition, outcome measures, animal models, fluid therapy and information technology needs: the Second International Consensus conference of the Acute Dialysis Quality Initiative (ADQI) group Crit Care 2004 8 4 R204 R212 15312219 10.1186/cc2872 PMC522841

[B7] Hoste EA Clermont G Kersten A Venkataraman R Angus DC De Bacquer D Kellum JA RIFLE criteria for acute kidney injury are associated with hospital mortality in critically ill patients: a cohort analysis Crit Care 2006 10 3 R73 16696865 10.1186/cc4915 PMC1550961

[B8] Mehta RL Kellum JA Shah SV Molitoris BA Ronco C Warnock DG Levin A Acute Kidney Injury Network Acute kidney injury network: report of an initiative to improve outcomes in acute kidney injury Crit Care 2007 11 2 R31 17331245 10.1186/cc5713 PMC2206446

[B9] Chertow GM Burdick E Honour M Bonventre JV Bates DW Acute kidney injury, mortality, length of stay, and costs in hospitalized patients J Am Soc Nephrol 2005 16 11 3365 3370 16177006 10.1681/ASN.2004090740

[B10] Kohli HS Bhat A Jairam A Aravindan AN Sud K Jha V Gupta KL Sakhuja V Predictors of mortality in acute renal failure in a developing country: a prospective study Ren Fail 2007 29 4 463 469 17497470 10.1080/08860220701260651

[B11] Arije A Kadiri S Akinkugbe OO The viability of hemodialysis as a treatment option for renal failure in a developing economy Afr J Med Med Sci 2000 29 3–4 311 314 11714013

[B12] Chugh KS Renal disease in India Am J Kidney Dis 1998 31 3 Ivii Iix 9506675

[B13] Vachvanichsanong P Dissaneewate P Lim A McNeil E Childhood acute renal failure: 22-year experience in a university hospital in southern Thailand Pediatrics 2006 118 3 e786 e791 16894011 10.1542/peds.2006-0557

[B14] Askenazi DJ Feig DI Graham NM Hui-Stickle S Goldstein SL 3–5 year longitudinal follow-up of pediatric patients after acute renal failure Kidney Int 2006 69 1 184 189 16374442 10.1038/sj.ki.5000032

[B15] Xue JL Daniels F Star RA Kimmel PL Eggers PW Molitoris BA Himmelfarb J Collins AJ Incidence and mortality of acute renal failure in medicare beneficiaries, 1992 to 2001 J Am Soc Nephrol 2006 17 4 1135 1142 16495381 10.1681/ASN.2005060668

[B16] Waikar SS Curhan GC Wald R McCarthy EP Chertow GM Declining mortality in patients with acute renal failure, 1988 to 2002 J Am Soc Nephrol 2006 17 4 1143 1150 16495376 10.1681/ASN.2005091017

[B17] Stevens PE Tamimi NA Al-Hasani MK Mikhail AI Kearney E Lapworth R Prosser DI Carmichael P Non-specialist management of acute renal failure QJM 2001 94 10 533 540 11588212 10.1093/qjmed/94.10.533

[B18] Metcalfe W Simpson M Khan IH Prescott GJ Simpson K Smith WC MacLeod AM Scottish Renal Registry Acute renal failure requiring renal replacement therapy: incidence and outcome QJM 2002 95 9 579 583 12205335 10.1093/qjmed/95.9.579

[B19] Hegarty J Middleton RJ Krebs M Hussain H Cheung C Ledson T Hutchison AJ Kalra PA Rayner HC Stevens PE O’Donoghue DJ Severe acute renal failure in adults: place of care, incidence and outcomes QJM 2005 98 9 661 666 16055475 10.1093/qjmed/hci109

[B20] Ali T Khan I Simpson W Prescott G Townend J Smith W Macleod A Incidence and outcomes in acute kidney injury: a comprehensive population-based study J Am Soc Nephrol 2007 18 4 1292 1298 17314324 10.1681/ASN.2006070756

[B21] Census population Age and gender profile (unrounded) 2011 Available from: http://www.kent.gov.uk/__data/assets/pdf_file/0005/12479/2011-Census-population-age-and-gender-profile.pdf

[B22] Lafrance JP Miller DR Defining acute kidney injury in database studies: the effects of varying the baseline kidney function assessment period and considering CKD status Am J Kidney Dis 2010 56 4 651 660 20673605 10.1053/j.ajkd.2010.05.011

[B23] Lewington A Kanagasundaram S Acute Kidney Injury Clinical Practice Guideline 2011 http://www.renal.org/guidelines/modules/acute-kidney-injury#sthash.n1u9bBah.dpbs 10.1159/000328075 21555903

[B24] Quan H Sundararajan V Halfon P Fong A Burnand B Luthi JC Saunders LD Beck CA Feasby TE Ghali WA Coding algorithms for defining comorbidities in ICD-9-CM and ICD-10 administrative data Med Care 2005 43 11 1130 1139 16224307 10.1097/01.mlr.0000182534.19832.83

[B25] Hux JE Ivis F Flintoft V Bica A Diabetes in Ontario: determination of prevalence and incidence using a validated administrative data algorithm Diabetes Care 2002 25 3 512 516 11874939 10.2337/diacare.25.3.512

[B26] Health and Social Care Information Centre Open Exeter: Online access to National Health Applications and Infrastructure Services (NHAIS) system 2014 Available at: https://nww.openexeter.nhs.uk/nhsia/index.jsp, 2014

[B27] Stewart J Findlay G Smith N Kelly K Mason M Adding Insult to Injury: A Review of the Care of Patients Who Died in Hospital With a Primary Diagnosis of Acute Kidney Injury (Acute Renal Failure) 2009 http://www.ncepod.org.uk/2009report1/Downloads/AKI_summary.pdf

[B28] Kolhe NV Stevens PE Crowe AV Lipkin GW Harrison DA Case mix, outcome and activity for patients with severe acute kidney injury during the first 24 hours after admission to an adult, general critical care unit: application of predictive models from a secondary analysis of the ICNARC case mix programme database Crit Care 2008 12 Suppl 1 S2 19105800 10.1186/cc7003 PMC2607110

[B29] Siew ED Ikizler TA Matheny ME Shi Y Schildcrout JS Danciu I Dwyer JP Srichai M Hung AM Smith JP Peterson JF Estimating baseline kidney function in hospitalized patients with impaired kidney function Clin J Am Soc Nephrol 2012 7 5 712 719 22422536 10.2215/CJN.10821011 PMC3338282

[B30] Siew ED Matheny ME Ikizler TA Lewis JB Miller RA Waitman LR Go AS Parikh CR Peterson JF Commonly used surrogates for baseline renal function affect the classification and prognosis of acute kidney injury Kidney Int 2010 77 6 536 542 20042998 10.1038/ki.2009.479 PMC2929703

[B31] Siew ED Peterson JF Eden SK Moons KG Ikizler TA Matheny ME Use of multiple imputation method to improve estimation of missing baseline serum creatinine in acute kidney injury research Clin J Am Soc Nephrol 2013 8 1 10 18 23037980 10.2215/CJN.00200112 PMC3531649

