# Supplementary material for: What is the real impact of acute kidney injury?
Source: BMC Nephrol. 2014 Jun 21;15:95. doi: 10.1186/1471-2369-15-95 (PMC4079645; doi:10.1186/1471-2369-15-95)
Supplement: Additional file 1: Table S1 — Primary diagnoses used in the analysis, by ICD-10 group. [file 1471-2369-15-95-S1.doc]

**Additional file 1: Table S1:** Primary diagnoses used in the analysis, by ICD-10 group.

| Elective admission |
| --- |
| Infectious diseases |
| Blood disease |
| Circulatory diseases |
| Digestive diseases |
| Ear diseases |
| Eye diseases |
| Genitourinary diseases |
| Musculoskeletal dis. |
| Nervous system dis. |
| Respiratory diseases |
| Skin diseases |
| Endocrine diseases |
| Health status factors |
| Poisoning/external |
| Mental disorders |
| Neoplasms |
| Other admissions |
